# OVERVIEW OF THE NEBRASKA GERIATRIC BEHAVIORAL HEALTH WORKFORCE SURVEY

**DOI:** 10.1093/geroni/igad104.1031

**Published:** 2023-12-21

**Authors:** Cecilia Poon

**Affiliations:** Nebraska Medicine/ UNMC, Omaha, Nebraska, United States

## Abstract

According to the US Census, more than 16% of Nebraska’s population is 65 and older. Many older Nebraskans reside in rural communities. Although clinicians in existing state-wide workforce surveys endorse working with older adults, it is unclear how frequently they are involved in geriatric mental health. To identify the needs of those who are providing mental health services to older Nebraskans, the Behavioral Health Education Center of Nebraska (BHECN) conducted an online survey of eligible licensed practitioners from August to December 2022. This presentation highlights the background characteristics of 193 respondents, nearly 40% of whom came from rural areas. Two-thirds identified either as licensed mental health therapists (38%), licensed clinical social workers (26%), or licensed psychologists (14%). More than a quarter did not provide direct clinical services to older adults. On average, respondents estimated devoting 18% of their time working with older adults. Respondents listed insurance restrictions, lower reimbursement rates, and limited specialized geriatric mental health training opportunities as major reasons that had kept them from serving older clients more frequently. These findings support BHECN’s ongoing efforts to further develop training and support, and advocate for potential policy changes to enhance the state’s geriatric behavioral and mental health workforce.

